# The downgrading of pain sufferers’ credibility

**DOI:** 10.1186/s13010-021-00105-x

**Published:** 2021-10-06

**Authors:** Mar Rosàs Tosas

**Affiliations:** Blanquerna School of Health Sciences at Ramon Llull University, C/Padilla 326, 08025 Barcelona, Spain

**Keywords:** Credibility, Communication, Epistemic injustice, Objectivity, Pain, Skepticism, Subjectivity

## Abstract

**Background:**

The evaluation of pain remains one of the most difficult challenges that healthcare practitioners face. Chronic pain appears to affect more than 35% of the population in the West, and indeed, pain is the most common reason patients seek medical care. Despite its ubiquity, studies in the last decades reveal that many patients feel their pain is dismissed by healthcare practitioners and that, as a result, they are denied proper medical care. Buchman, Ho, and Goldberg (J Bioethic Inq 14:31-42, 2017) point to this phenomenon as a form of “epistemic injustice”: an unfair and harmful downgrading of credibility affecting some individuals and groups, which prevents them from receiving appropriate and beneficial medical care.

**Methods:**

By exploring the existing literature on this downgrading of patients’ credibility written by healthcare professionals and scholars in medical humanities, I identify and examine the reasons patients are often not believed about their pain and why healthcare is too-often unhelpful or hurtful to people presenting with chronic pain. I also explore to what extent it is possible to forge an alternative epistemological model.

**Results:**

I suggest that most of the causes of this downgrading of patient’s credibility result from either the *difficulty in communicating pain* or the widespread belief that pathology is always the result of *objective tissue damage*. I examine whether pain has to be effectively communicated and have an objective cause in order for it to be deemed credible. In the end, I argue that in the case of pain, both communication and objectivity are highly problematic.

**Conclusions:**

I conclude by suggesting that, although alternative epistemological models might be impossible to build, believing patients has both moral and clinical benefits, and this warrants further research.

## Background

The evaluation of pain remains one of the most difficult challenges facing healthcare practitioners. Chronic^1^ pain affects more than 35% of the population in the West ([3]: 24). And, indeed, pain “is the most common reason a person will seek medical help” ([1]: 490). Despite pain being such a common experience, many pain sufferers –particularly those suffering chronic pain- report that they are not “being believed” and that, as a result, they are not provided the attention and help they think they need and deserve [1, 3, 4]. This skepticism on the part of medical practitioners, though, contradicts the certainty of one’s own pain, as Scarry [5] recalled in her influential work *The Body in Pain*: “‘having pain’ may come to be thought of as the most vibrant example of what it is to ‘have certainty,’ while for the other person it is so elusive that ‘hearing about pain’ may exist as the primary model of what it is ‘to have doubt’” ([5]: 4).

In the past several decades, the dismissal of others’ pain has been extensively studied by healthcare professionals and scholars in medical humanities^2^ hailing from anthropology and sociology of health, history, philosophy, bioethics, psychology, cultural studies, and literary studies, using a wide range of research methods from ethnographic work to quantitative studies and philosophical analysis [1–20].

A common point of agreement across these studies is that “doubt and skepticism regarding the existence, scope, and legitimacy” of others’ pain “flows from all participants in cultures of pain: healthcare practitioners, caregivers, fellow pain sufferers, and even pain sufferers themselves [12]” ([10]: 34). Members of all these groups, then, seem to be experiencing what Buchman, Ho and Goldberg [10] label “epistemic injustice”: a downgrading of credibility. In Buchman et al.’s words, epistemic injustice is:

a type of harm that is done to individuals or groups regarding their ability to contribute to and benefit from knowledge. In particular, it happens when a prejudice causes a hearer to give less credibility to a speaker’s testimony and interpretations than they deserve. Since there are major inequities in the prevalence, treatment, and outcomes for chronic pain across race, ethnicity, gender, and class, epistemic injustice may also be associated with distrust as well as broader patterns of stigma and social injustices ([10]: 32).

In epistemic injustice, ethics and epistemology are interwoven -epistemic injustice is a harmful *ethical* attitude that, by adhering to a certain *epistemological* model, does not acknowledge that which nor those whom indeed deserve acknowledgement.^3^ This form of injustice not only affects pain, of course; rather, it is at play in a wide range of social, economic, political, and cultural phenomena. Buchman’s, Ho’s and Goldberg’s work has consisted of applying the notion of “epistemic injustice”, coined by Fricker [21], to the exploration of a phenomenon specific to health –the experience of pain sufferers.

## Methods and goals

The goal of the present paper is twofold. First, to identify and examine the reasons why patients are often not believed about their pain and why healthcare is too-often unhelpful or even hurtful to people presenting with chronic pain. This examination closely follows existing literature on the downgrading of pain sufferers’ credibility written by healthcare professionals and scholars in medical humanities. The second goal is to explore to what extent it is possible to forge an alternative epistemological model. In this sense, this paper complements Buchman’s, Ho’s, and Goldberg’s analysis. Their cogent account and critique of this phenomenon insists on the need to counteract the epistemic injustice affecting pain sufferers with what they label “epistemic justice” and “epistemic humility”. It is my contention, however, that their attempt to describe these alternative attitudes comes up short because they fail to identify and examine two fundamental epistemological difficulties at the heart of skepticism towards others’ pain. The first epistemological difficulty concerns *communication* –can pain be effectively communicated? And must pain be effectively communicated in order for it to be deemed credible and properly evaluated? The second difficulty is deeply interwoven within the first: the idea that communication might (or might not) be effective presupposes a commonly held distinction in biomedicine: *that of an objective reality* –that the healthcare practitioner tries to grasp- *and its subjective experience* –or the patient’s account, through which the objective reality manifests. In the case of pain, does this distinction hold? I will examine these two issues in order to explore the possibility of an alternative epistemological model in which patients’ pain would not be dismissed, which Buchman, Ho, and Goldberg envisage, but do not specify.

The paper will unfold as follows. In Section 1. Consequences of the downgrading of pain sufferer’s credibility, I will identify and describe the wide range of consequences stemming from the downgrading of pain sufferers’ credibility, thus demonstrating the scope of the problem. In Section 2. Causes of the downgrading of pain sufferers’ credibility, I will enumerate and explore the causes of the phenomenon, while in Section 3. Attempts to overcome epistemic injustice against pain sufferers, I will critically examine some attempts to overcome them. At that point I will identify the two aforementioned epistemological difficulties, which will be addressed, respectively, in Section 4. Can pain be properly communicated? and Section 5. Does the distinction objective-subjective hold in the case of pain?. By way of conclusion, I will suggest that, although these two difficulties might be unsurmountable and, therefore, it might not be possible to build an alternative epistemological model, patients can still expect to “be believed” and benefit from it.

### Section 1. Consequences of the downgrading of pain sufferer’s credibility

A review of the literature in medical humanities, with regard to the lack of credibility affecting many people experiencing pain, reveals that this discredit has far-reaching and harmful consequences. A first consequence of this form of epistemic injustice is a *lack of diagnosis*.^4^ If no objective cause is identified following thorough diagnostic testing and imaging, many patients are told either that they are “fine” or that it is all “in their head”. Having exhausted all possible objective causes, they are then referred to a psychiatrist (Scull [22]: 186–188). Not receiving a diagnosis also means not receiving the “relief” ([23]: xv) and validation a concrete diagnosis might offer. When symptoms are presented as invalid, patients tend to feel a “deep distress” [14] often accompanied by either shame or guilt, a common experience that has been extensively studied ([24]: 30–32; [25]; [26]: 9–11; [27]; [28]: 18, 59), and which lead Diamond to coin the expression “blame-the-patient-philosophy” ([29]: 10).

A second immediate consequence of this epistemic injustice is *a sense of humiliation*. The patient’s narrative is often perceived with suspicion and is marginalized when it does not coincide with the official, medical narrative since, according to the modern understanding of illness, “[t]he story told by the physician becomes the one against which others are ultimately judged true or false, useful or not” ([23]: 5). If, despite all efforts to be credible ([10]: 37), no cause for the patient’s described sensations can be identified, the patient is doubted ([3]; [30]: 74) and perceived as a non-reliable narrator ([31]: 238). A third consequence of not acknowledging the legitimacy of patients’ pain is that it further complicates the already *challenging task of drawing a new map to navigate the new territory* exposed by the affliction. As sociologist of health Arthur W. Frank [23] studied, when a serious pathology irrupts, life is disrupted and old maps are no longer useful. Acknowledgment from family, friends, and medical authorities is fundamental to being able to forge new maps or stories “to repair the damage that illness has done to the ill person’s sense of where she is in life, and where she may be going”; to help her with “redrawing maps and finding new destinations” (Frank [23]: 53). When instead, patients are told that nothing is wrong, fashioning a coherent or meaningful narrative becomes an arduous task, in particular because family, friends, and healthcare practitioners tend to encourage patients to “just be positive”, that is, to build what Frank [23] labeled a “restitution narrative”, which psychotherapist and narratologist Conway [11] recast as a “triumph narrative”: a narrative attentive to “good signals” that silences negative thoughts and that celebrates illness for bringing about good things. As Conway astutely noted, “our cultural insistence on triumph can result in harm to patients, contributing at times to a refusal on the part of caretakers to hear reports of pain” ([11]: 8).

*The denial of proper assistance* –analgesics and other treatment- is a fourth consequence of the downgrading of credibility. For example, as Biro recalls, a study conducted by the US Department of Health and Human Services revealed that “more than 50 percent of cancer patients didn’t receive adequate analgesia, and about 25 percent of them were estimated to die in severe, unrelieved pain” ([8]: 35). Indeed, when asking “why weren’t effective ways to blunt acute pain introduced before the mid-nineteenth century?”, Bourke goes so far as to argue that it had to do, at least in part, with the “illegitimacy of feeling pain” ([3]: 272–275).

A fifth consequence is that patients are often *denied sick leave*. As the “sick role”^5^ dictates, because of their inability to continue performing certain professional and social activities, the sick are granted particular rights, such as having the right to be taken care of and from the right to miss work without repercussions, sometimes accompanied by a “right to a disability pay” ([33]: 142). When sick leave or disability pay is denied, as is often the case for patients with chronic pain, their experience of being a burden worsens.

The sixth and final consequence is the *pilgrimmage* that many patients find themselves forced to make in order to find a practitioner who does not dismiss them and who instead helps them make sense of their experience.^6^ On some occasions, these professionals are found within conventional medicine. In others, the pilgrimage becomes a one-way trip to the terrain of so-called alternative medicine –with the risks it might entail.

In short, the downgrading of credibility has deleterious effects in several dimensions –economic, physical, social, existential. The next sections offer a description and analysis of the causes of this phenomenon that will allow us to examine the possibility of an alternative epistemological model that would not harm patients in these ways.

### Section 2. Causes of the downgrading of pain sufferers’ credibility

Medical humanists have addressed the causes of discrediting pain sufferers through a wide range of methods -ethnographic work, quantitative studies, historical explorations, bioethical analysis, and discourse analysis. My review of this literature reveals that the phenomenon owes to five main causes, which might present either separately or altogether. The goal of this section is to offer a panoramic view of these causes and suggest that these factors hinder patients’ credibility for one fundamental reason –in many cases of chronic pain, the pain signal may not be coming from a specific peripheral location, impeding any attempt to identify objective tissue damage.

The first cause affects those whose expressions of pain and illness narratives tend to be considered less trustworthy because of *prejudice or stigma surrounding the group they belong to* ([10]: 37). That is the belief that some groups do not experience pain in the same way,that they have less emotional control, tend to exaggerate more,^7^ or simply lie to gain access to benefits like disability pay. All recent histories of pain concur on the point that *which* groups are taken seriously varies across time and place ([2, 3]; Boddice 2014).^8^ But “the young, female, poor, and minority ones” have tended to “face much higher risks of being under-treated for pain than other groups” ([3]: 292–294), while other groups, as long as they master the strategies of “claiming power” appropriate to cultural context, manage to be more convincing to the audience ([10]: 37; [35]: 183).

In a way, this tendency to disregard the illness narratives of a particular group implies that many healthcare practitioners do not listen well enough, a claim showing up over and over again in the medical humanities literature for the last three decades, which brings us to the second cause of downgrading credibility. When healthcare practitioners don’t listen well, they will likely fail to accurately diagnose their patients, jeopardizing any possibility of a successful treatment plan. In a word, they *lack “narrative competence”.* If they had it, they would be practicing “narrative medicine”,^9^ to use the famous term coined by Charon [24].

The third cause, according to some scholars, is that *pain cannot be effectively expressed and communicated*. Some scholars go as far as to claim that pain is the least communicable experience of all.^10^ Others argue that only some aspects of pain are unsharable ([31]: 235). According to all of these scholars, this difficulty is due to the fact that we lack words–the experience of pain cannot be encapsulated in language; indeed, it surpasses it [8]. First, because “seriously ill people are wounded not just in body but in voice” (Frank [23]: xx). And, second, because of *the limited nature of language* -however encompassing it might be, it is ultimately inadequate. Therefore, pain resists language ([31]: 235). It exists, in part, outside of language. This stance echoes Derrida’s post-structuralist, epistemological framework, in which words never really grasp the signified, since there is no outside of language ([37]: 73; [38]: 365). However, for these medical humanists, the reason pain cannot be grasped by language is precisely that part of it *does* exist outside of language.

Other scholars in the medical humanities as well as pain sufferers in their own memoirs maintain that the reason that pain cannot be properly expressed stems not only from the nature of *language*, but also from the nature of *pain* –pain destroys language. Scarry’s thesis states: “Physical pain does not simply resist language but actively destroys it, bringing about an immediate reversion to a state anterior to language, to the sounds and cries a human being makes before language is learned” ([5]: 4). Over the last few decades, Scarry’s view has enjoyed wide acceptance and lasting influence among medical humanities scholars ([8, 10, 31]; [39]^11^), although most of them link her idea with the claim that *some* forms of language are indeed not destroyed by pain, and thus actively encourage patients and healthcare practitioners to turn to these forms –mainly, metaphors [3, 6, 15, 40].^12^ Indeed, one could argue that most language used in medicine is metaphorical (e.g., can’t catch my breath, elephant sitting on my chest, I have a frog in my throat).

In short, whether a result of the limited nature of language or of the destructive character of pain, patients “find themselves tongue-tied” ([8]: 13) both when they try to describe their *type* of pain as well as its *intensity*.

The *difficulty –if not impossibility- of quantifying pain* is the fourth cause usually given by healthcare professionals and medical humanists concerning epistemic injustice against chronic pain sufferers ([3]: 266). Two people experiencing the same type and amount of pain could rate it differently on a scale of 0–10. This is because the number they choose depends on what they imagine as the highest possible level of pain and also on their ability to measure or rate their pain against the so-called maximum pain level.

The last cause of epistemic injustice I would like to draw attention to is that pain cannot be used as a “cognitive tool” ([2]: 95) in those cases in which there seems to be *no clear connection between the (subjective) experience of pain and an (objective) cause* that could account for that pain. Medical humanists have expressed it with different formulas: for Scarry, this type of pain is “objectless” ([5]: 162); for Biro, it “lacks intentionality” ([8]: 39); for Good, it “resists localization” ([40]: 39). The most commonly used formula in recent years is “a lack of correlation” between the (subjective) experience of pain and the existence of an (objective) tissue damage ([2]: 8, 88–89, 108, 166; [8]: 41; [10]: 33–34). In Bourke’s words: “[t]here is no necessary and proportionate connection between the intensity of tissue damage and the amount of suffering experienced” ([3]: 8). And, in Biro’s words, “pain sets up an ontological divide. There is the reality of the person in pain and the reality of those on the outside. Because there is no way to verify the pain of another, no objective test even in our age of MRIs and PET scans, these radically different realities are unbridgeable” ([8]: 32). This does not necessarily mean the healthcare practitioner considers the pain “invalid”, but, as long as that form of pain cannot be “seen” in a test, it is useless for diagnostic purposes.

This phenomenon is not new. Hydén [25] suggested that this dismissal of patients’ personal accounts in favor of demonstrable tissue damage dates back to the turn of the twentieth century. Others, though, have argued that it dates back to French physician Bichat (1771–1802), considered the father of modern histology, and his disciple Broussais (1772–1838). As Engelhard put it, with them, “[t]he accent of medical attention (…) shifted from patients and their complaints to the organs and bodies of patients” ([33]: 151). Or, in Bourke’s words, from the eighteenth century to the present, “pain narratives became mere ‘noise’, serving little diagnostic purpose” ([3]: 132) except when they could contribute to the “localization of a lesion or a pathological state” ([3]: 133).

Most historians of medicine consider the logic informing Bichat’s approach to be indebted, in turn, to Andrea Vesalius’ treatise *De humani corporis fabrica* (1543), which presents the human body as a “mechanism” or machine; if there is an affliction, there must be an underlying, identifiable cause ([31]: 227; [42]: 23; [43]: 156; [44]: 84). This logic has also been attributed, at least in part, to Descartes [45]–not only to his distinction between the res cogitans and the res extensa ([46]: 30), but also to his reflection, in the VI Meditation (originally published in 1641), on whether a sick person can be compared to a broken clock ([30]: 59).

The emergence and implications of this “mechanistic model” of the human body were best explored by Foucault [47]. For him, Bichat, in creating pathological anatomy, opened up a new way of looking at the human body, the so-called “anatomo-clinical gaze” –pathology became visible and, therefore, utterable. Thus the modern understanding of disease was inaugurated. Toombs, however, points out the weaknesses of this new model: “this especially mechanistic model includes little, if anything, of the patient’s experience of illness. Rather than being the central focus, the patient’s subjective experiencing is relegated to the periphery. It is the X-rays, the laboratory studies, the pathology reports –and not the lived experience- which are taken to constitute the central phenomenon of disease” ([31]: 227–228). It is an approach that lays the emphasis on geography rather than on history, in Moscoso’ words ([2]: 88).

In short, the ultimate reason why many patients feel that “physicians do not trust (hence, hear) the human voice” and try to bypass it “as quickly as possible so that they can get around it to the physical events themselves” ([5]: 6–7) is the popularization of this model of “mechanical objectivity”, which is still prevalent in medicine today ([10]: 34).^13^ Bourke draws attention to the fact that the definition of pain proposed in 1976–77 by the International Association for the Study of Pain (IASP) reflects this Cartesian divide ([3]: 12) and the logic behind the “medical gaze”: pain is defined as “an unpleasant sensory and emotional experience associated *with actual or potential tissue damage*, or described in terms of such damage” ([3]: 10). Later definitions of pain by the International Association for the Study of Pain also described it as a “warning of actual or potential tissue damage” [49].

In most cases of chronic pain, “any initiating injury has usually resolved” ([1]: 490). According to the IASP definition, then, these patients cannot legitimately claim that they are in pain. Hence Buchman’s conclusion: “[p]ain frustrates dominant models of mechanical objectivity within biomedical cultures. It evades the clinical gaze that stands as a powerful truth-making criterion in such cultures” ([10]: 37).

### Section 3. Attempts to overcome epistemic injustice against pain sufferers

Could this epistemological model be subverted or, at least, modified so that chronic pain sufferers should not have to bear the negative socioeconomic, existential, and physical consequences of their pain being dismissed?

In response to the problem, the medical community has launched efforts to design strategies to quantify and objectify pain, as is the case of the above mentioned “pain scale”. However, with its limited capacity to grasp the *intensity* of pain, and its incapacity to grasp the *type* of pain, this practice that “reduces language to the numbers one through ten (…) merely create[s] an illusion of precision” ([6]: 59). This also happens with the scale of 21 pain intensity units designed in the mid-twentieth century by Hardy, Wolff, and Goodell ([2]: 107). The Faces Pain Scale has not been much of an improvement, although healthcare providers rely much more on it ([8]: 13).

Among the strategies to help communicate and objectify pain, the McGill Pain questionnaire occupies a prominent place. Despite the merit of this lengthy list of words to describe pain created in the 1970s, the questionnaire faces inevitable shortcomings. First, that “[p]atients select from a limited range of words, some of which may not be familiar” ([6]: 49). And, second, “the effect [these sort of questionnaires] have had on how patients narrate (and are *taught* to recount) their distress: (…) in practice, they have tended to constrain languages of pain” ([3]: 152–153).^14^ Bourke goes as far as to claim that the questionnaire “helps to create the phenomenon it purports to measure” ([3]: 154).

Brain imaging is another strategy that is being used to contribute to the objectivation of pain. In recent years, it has made it possible to visualize *objective* tissue damage in the brain of patients who claim they are in pain, but for no apparent reason. For these patients, neuroimaging provides relief and reduces guilt. Nevertheless, this has only been granted to a small group of patients in pain. What brain imagining shows, in most cases, is the *brain activity* –not tissue damage- that can be observed when a person experiences pain. Patients often experience relief when brain imaging shows there is something “going on” (–you see? I am not making it up!). However, this relief comes from a misinterpretation of what brain imaging actually shows: it reveals a neurological response to pain, not a *source* of pain.^15^

Do all of these efforts, shortcomings aside, help subvert the epistemological model that downgrades pain sufferers’ credibility? Unfortunately, not really. Indeed, these efforts are trapped in the following contradiction: insofar as they seek the objectivation of pain, they reinforce the very logic that ignores and marginalizes the voices of many people in pain, because they continue to discredit subjectivity as a source of reliable information. They perpetuate, therefore, the epistemic injustice against pain sufferers [10, 13].

Have there been attempts to forge alternative models that either correct or complement the epistemological flaws of the predominant biomedical model? Definitely. The most influential one is probably [51] proposal to perform a shift from the biomedical model –mainly biomechanistic, and indebted to Bichat and Vesalius- to what he labeled a biopsychosocial model. This paradigm, which is being implemented across the globe, does not question, however, the need for *objective causes* inherent to the biomedical model, but rather complements it by granting importance -both in the diagnosis and in the treatment- to the patient’s experience and context.

Buchman et al. [10] also tried to dismantle the biomedical model. As explained above, they did so by proposing that practitioners help patients with what Fricker [21] labels “epistemic humility”. Epistemic humility requires “healthcare practitioners to critically evaluate the implicit assumptions inherent in the anatomo-clinical method, especially as this method categorically privileges certain kinds of knowing over others (e.g., the objective MRI results over the subjective patient testimony)” ([10]: 38). Briefly, Buchman’s epistemic humility requires i) seriously taking into consideration the patient’s subjective narrative^16^ and also, ii) assuming that biomedicine is not, in practice, objective –ultimately, diagnostic and therapeutic processes are social and political processes ([10]: 33). According to them, the practice of epistemic humility would lead to epistemic justice, “a hybrid epistemic–ethical virtue that a hearer possesses in order to counterbalance the impact of prejudice in their credibility judgments” ([10]: 38).

Buchman, Ho, and Goldberg’s analysis, though, ends at this point. They do not develop in detail how subjectivity could be incorporated more into diagnostic and therapeutic processes. And, therefore, they do not meet two of the fundamental challenges that, in my opinion, any attempt to forge an alternative epistemological model must confront and endeavor to overcome. The first challenge, which will be addressed in Section 4. Can pain be properly communicated?, concerns *communication*. Can pain be *effectively, successfully* communicated? And does it need to be effectively communicated in order for it to be credible? The idea that communication might (or might not) be effective seems to presuppose a commonly held distinction in biomedicine: the *distinction between an objective reality* that the healthcare practitioner tries to grasp –*disease*, studied by biomedicine- *and the subjective experience* of the patient, through which the objective reality manifests –*illness*, studied by anthropology.^17^ But, in the case of pain, does the distinction hold? This second challenge will be dealt with in Section 5. Does the distinction objective-subjective hold in the case of pain?.

In spite of Buchman’s and our keenness to grant a prominent role to subjectivity in diagnostic and therapeutic processes, it should not be forgotten that objectivity and causality are, after all, pillars of biomedicine. In other words: this distinction might not be so easy to overcome.

### Section 4. Can pain be properly communicated?

In Section 2. Causes of the downgrading of pain sufferers’ credibility, I argued that one of the causes of the downgrading of pain sufferer’s credibility pointed to by most medical humanists is that pain cannot be effectively communicated. But among their ranks there are also more optimistic voices. The goal of this section is to classify these optimistic voices into three groups, describe their positions and point out that their optimism rests upon a limited understanding of what communication is. This will lead us to reconsider the relationship between accurate description of pain and the credibility of patients in pain.

The first group includes those who maintain that pain is unsharable, but talking and *writing about it might bring about positive side effects*. Conway [11] has extensively studied how this belief is embedded in a number of literary works from the nineteenth century through the 21^st^. In these texts, she sees that, although the descripiton of pain is never accurate, and, therefore, its communication never succesful, one might still “feel the need” ([11]: 82) to keep describing it and writing about it, and this might have great effects: it might “heal” ([11]: 3); it might help the “person make peace with his or her situation” (120) and it might “create a space in which the most devastating aspects of the experience of serious illness and dying can be articulated, reflected upon, and shared –the loss of control, ruptures in the self, disruptions in the life story, and questions of meaning in the face of personal annihilation” (9). In short, the “failure of literature” of fully expressing illness “paradoxically allows us as readers to approach the ground of desolation” (Conway [11]: 16).

The second group includes those who, despite claiming that pain is inexpressible, suggest that there might be *ways to overcome this inexpressibility*. It includes approaches such as Biro’s [8],^18^ Jurecic’s [6],^19^ and Lunati’s [41]. For them, overcoming the feeling of inadequacy between the experience of pain and the words to refer to it requires *using proper linguistic means* -hence the proliferation of creative writing classes for patients in pain. Often, metaphor is considered the linguistic medium par excellence to convey pain and illness. Such is Biro’s case, and, to a lesser extent, Jackson’s [15],^20^ Bourke’s [3],^21^ and Lunati’s [41].^22^ At the other end of the spectrum, though, there are those following Sontag’s [28] influential work on illness and metaphor, who hold that illness needs to be freed from metaphor because its use adds certain connotations to the existing condition that further harm the sick. In any case, most of these proposals take for granted that ordinary language and metaphor can be clearly distinguished, while philosophy of language insists on the blurriness of the lines that appear to separate them.^23^

The third group includes those medical humanists who hold that the experience of pain is communicable *for epistemological reasons* –and not merely whether one has the right metaphors or sufficient rhetoric skills. Their point is that pain is not as *private* as it might seem at first glance, and, as such, might not be that *subjective*. Among such scholars, Bourke [3] and Biro [8] stand out for their insistence on the *public* –and therefore sharable, communicable- side of pain. Interestingly, they both turn to Wittgenstein’s *Philosophical Investigations* [53] to argue this.^24^ Biro explains that Wittgenstein questions the widespread belief according to which “each of us has a richly meaningful private world” that is unsharable ([8]: 49). If that were the case, people within communities would not be able to understand each other. For Wittgenstein, Biro goes on, language “is a practice that involves many people and that must be anchored in a public, shared space where it can be agreed upon, negotiated, and renegotiated over time. Otherwise it would be useless” ([8]: 53). And, in order to stress his idea that our seemingly private world is not that private, Wittgenstein picks *precisely* the experience of pain, which, at first, might seem to be one of the most radically private experiences. Bourke puts it as follows: “To have meaning, Wittgenstein concluded, words for feeling states like pain must be inter-subjective and able, therefore, to be learned (…). Although pain is generally regarded as a subjective phenomenon (…), ‘naming’ [pain] occurs in public realms” ([3]: 6). In short, Wittgenstein reveals that language bridges the divide between the private and the public realms. Thus, some medical humanists turn to this line of argumentation to insist on the idea that descriptions of pain might illuminate the experience in ways that we can all understand.

For these three groups of optimistic scholars, language can grasp the experience of pain, at least in part. Why, then, do patients with chronic pain so often claim that they are not being understood? For some philosophical frameworks, the words uttered by an individual, however accurate they may be, never mean the same thing to the speaker and her audience. This is the case for Derrida, whose epistemological framework has been highly influential in contemporary Continental philosophy: he writes that nothing can be successfully communicated, not even the word communication itself, and thus contradicts Austin’s theory of successful speech acts ([38]: 367–384). It is also the case in Zizek’s epistemological framework, inspired by Lacanian psychoanalysis: language does not *represent* reality, but *digs a hole* in reality, making it inaccessible and, therefore, unsharable ([54]: 122).

But the reasons that an audience does not fully understand another’s personal account of pain might not be only epistemological. They are often psychological and sociological. For communication to be successful there must be a person who *listens* and *understands*, and this not only depends on his or her knowledge of the linguistic code; it also depends on a healthcare setting allowing time for these types of medical encounters; on having the interest and skills to help patients develop their accounts of pain; on sharing a common cultural background; and on having had similar experiences. That is obviously something that theory of communication has studied at length [55]. À propos of illness narratives, Jurecic puts it as follows: “the primary problem they [sick people] face is not how to find language for pain, but rather how to make readers receptive to stories of pain. (…) [W]ho will listen and what will they hear?” ([6]: 44).

In short: even if the experience of pain could be properly described –something which, as pointed out, not all epistemological frames grant-, this does not automatically result in the audience understanding it. It takes two to communicate: an articulate speaker and a receptive listener. The latter is a consideration these three groups of optimistic voices fail to make.

Does this mean, then, that accounts of pain cannot be credible or valid? Does this imply, then, that the downgrading of credibility affecting pain sufferers is unavoidable? For some of the medical humanists mentioned so far, what renders pain narratives credible is their accuracy, but, for others, it is something else -whether they point to *objective* perceptions with *objective* causes.

### Section 5. Does the distinction objective-subjective hold in the case of pain?

For a perception to be objective, what is being perceived needs to appear to the individual without any trace of him or her, that is, in exactly the same form as if it was not being perceived by him or her. By contrast, a perception is subjective when what appears bears the trace of the individual who perceives it. This trace might be due to a wide range of factors -i.e., the angle from which one perceives the thing itself, one’s feelings, one’s prejudices, one’s sensorial apparatus or one’s previous experiences. Within this epistemological frame -for centuries, the Western traditional frame-, subjectivity is considered to distort perception and, therefore, is dismissed as a source of reliable information. The tendency^25^ to dismiss the pain sufferers’ narratives that do not point to objective perceptions and objective causes is a side effect of applying this epistemological framework to the realm of illness^26^ –which has its history, as explained in Section 2. Causes of the downgrading of pain sufferers’ credibility. In recent years, medical humanists specializing in pain have addressed this challenge. In what follows, I will describe and analyze the two most common strategies to try to solve it. I will argue that the inability to provide an alternative solid frame might suggest that the distinction objective-subjective, while problematic, might also be unsurmountable. I further argue that, despite this, *subjective accounts of pain might still be credible*.

The first group includes those who maintain that subjective accounts of pain need to be carefully listened to and believed because, one way or another, they offer valuable information that might contribute to a diagnosis and/or to design a proper treatment plan. For these defenders of subjective narratives, their value lies in their capacity to shed light on the *objective* dimension of pain. There is no questioning, then, of the traditional epistemological model. This position is visible across most of the works we have mentioned so far, those conducted both by healthcare professionals and by medical humanists (in particular, [8, 24]; [35]: 52, 148).

A small number of medical humanists, though, hold a different view that does question the traditional epistemology. Bourke, with her history of pain [3], is perhaps the author who has made the biggest effort to dismantle the distinction between subjectivity and objectivity in the case of pain. Her approach is partly inspired by the explanation of pain provided by Dr. Peter Mere Latham, a physician born in London in 1789, who wrote a number of texts on bodily agony: “Anyone claiming to be ‘in pain’ *is* in pain”, wrote Latham ([3]: 3). Accordingly, then, the person who claims being in pain should automatically be rendered credible.

For Bourke, as explained above, pain *is not an unsharable subjective experience*. Yet, at the same time, for her, pain *is not an objective entity* outside of one’s cultural background. Bourke’s understanding of pain, then, neither requires an *objective cause* behind the pain nor holds pain as an *objective reality*. In short, Bourke’s point is that *pain is only experienced as pain within a cultural framework*. This is why her “definition is skeptical, therefore, of any account that claims that pain is simply a sensual response to noxious stimuli” ([3]: 13). In this sense, Bourke aligns with two of the best-known histories of pain ([2]: 2–8; [7]). They all agree that pain is inherently sociocultural; that, “[f]rom the moment of birth, infants are initiated into cultures of pain” ([3]: 17).

Bourke is questioning, then, both the distinction nature/culture and objective/subjective, which explains her decision to use “the terms pain and suffering interchangeably”, because pain is usually considered an objective, natural reality and suffering its subjective experience within a given culture ([3]: 24).

Despite her insistence on deconstructing these dichotomies in the case of pain, these distinctions are still at play in her book. In the introductory pages, she seems to claim that pain is *fundamentally* -or solely- sociocultural; that there is no pain beyond culture. And she claims the same in her concluding remarks: “pain does not emerge naturally, from physiological processes, but in negotiation with social worlds (…). There is no pain-entity independent of the way it impinges on people’s being-in-the world” ([3]: 300–301). Historian of pain Moscoso appears to make a similar claim when he states that pain “does not exist outside its dramatic elements” and “differ[s] from one context to another. It is simply not the same sensation. There is no emotional reality out *there* than can be reinterpreted in accordance with cultural location or historical moment” ([2]: 35). However, Bourke, in the rest of the pages of her book, seems to hold a much less radical position: that pain is a reality in and of itself -which, initially, she denies- and that we experience it, express it and react to it depending on our sociocultural background.^27^

Perhaps she offers this second view in spite of herself, because the first view is extremely difficult, or impossible, to sustain. Our goal is in no way to criticize Bourke’s rigorous work, but rather to point out that even serious attempts to dismantle the distinction objective/subjective in the case of pain are not totally successful.

If the downgrading of pain sufferers’ credibility is the result, at least in part, of this distinction, does the failure to dismantle it indicate that this form of discredit might be impossible to overcome? For pain sufferers to be credible, does pain need to have an objective cause and/or to be an objective reality? In our view, no, it does not. First, because some objective causes might elude identification with current means. Second, because, as just discussed, the distinction objective/subjective is problematic in the case of pain.^28^

## Concluding remarks

Let’s resume the trajectory that led us here. The first goal of the present paper was to identify and examine the reasons why, according to the existing literature on the downgrading of credibility of many patients with pain by healthcare professionals and also within the medical humanities, sometimes patients are not believed about their pain and healthcare is too-often unhelpful or hurtful to people presenting with chronic pain.

After having examined the causes (Section 2. Causes of the downgrading of pain sufferers’ credibility) and consequences (Section 1. Consequences of the downgrading of pain sufferer’s credibility) of this phenomenon, I have turned to the second goal—to explore to which extent it is possible to forge an alternative epistemological model that does not marginalize subjective accounts of pain as a source of reliable information. I have argued that, to do so, we need a better understanding of two of the current conditions of possibility of believing patients in pain –that they succeed at *communicating* their experience (Section 4. Can pain be properly communicated?) and that said experience has an *objective* cause or might be considered *objective* (Section 5. Does the distinction objective-subjective hold in the case of pain?). I hope to have demonstrated that the epistemological framework supporting the modern understanding of illness is extremely limited, especially in the case of chronic pain (Section 3. Attempts to overcome epistemic injustice against pain sufferers, Section 4. Can pain be properly communicated? and Section 5. Does the distinction objective-subjective hold in the case of pain?). But, at the same time, I hope that the discussion on the limits of such an epistemological model has rendered manifest that overcoming this epistemological framework is a highly problematic exercise, if even possible.

Can the expression of pain still be credible or valid then? Yes, it can. It suffices to believe that the patient is not lying. Some patients lie, sure. And this is a point that cannot be ignored. According to certain medical humanists, it is plausible to believe that some patients lie because of the advantages that being ill might afford them. Parsons [32] already examined the “sick role” -the sick are granted special rights and are exempted from certain duties. In short, some patients might lie as a way to achieve “secondary gains” [56, 57].^29^

What are the risks of believing patients who lie? Healthcare practitioners tend to be afraid of granting credibility to false accounts of pain for the following reasons: i) patients might be prescribed drugs they do not need and which have significant side effects, ii) patients will want tests and further medical appointments they do not need, with the economic burden this implies for any healthcare system, iii) patients might use the medical acknowledgment of their pain to earn disability pay they do not deserve (achieving a secondary gain).

The issue of patients lying is, therefore, not minor. However, it is extremely difficult, if possible, to study whether and how many patients who claim to be in pain are actually lying. Interestingly, the reason why this exercise proves difficult, if possible, is the same reason why several claims of pain are not regarded as valid: because it is not always possible to find objectively-viewed causes of certain forms of pain. Therefore, whether and to what extent patients lie may be impossible to decipher.

Potential dishonesty is not the only reason many patients in pain aren’t being believed. As examined in Section 2. Causes of the downgrading of pain sufferers’ credibility, this disbelief is also due to i) prejudices surrounding certain groups, ii) healthcare practitioners’ lack of narrative competence, iii) the difficulty of describing pain, iv) the difficulty in identifying underlying tissue damage. In my view, while the risk of patients lying might obscure their credibility, these four additional reasons should not, as argued throughout this paper. In fact, one of the raisons d’être of this paper is precisely to claim that patients who claim to be in pain deserve to be relieved of the shadow of suspicion –even if some of them lie- because doing otherwise subjects them to the heavy and unnecessary burden studied in Section 1. Consequences of the downgrading of pain sufferer’s credibility. In other words, while believing the patients who lie might have the aforementioned negative consequences, the positive impact of believing those that do not lie are far-reaching. As Frank [23] famously wrote: “The need to honor (…) stories is both moral and clinical.”^30^

On the other hand, the truth is that many patients in pain are routinely believed by healthcare professionals, which might be seen in a number of decisions and gestures.^31^ Interestingly, these patients are believed i) despite the risk they might be lying and ii) despite their cases not fitting the epistemological model I have examined (Section 4. Can pain be properly communicated? and Section 5. Does the distinction objective-subjective hold in the case of pain?). Why are they being believed, then? And which epistemological models –probably used unconsciously, in most cases- sustain these gestures?

The end of the present inquiry leaves us with these important questions, which remain to be answered and warrant further research. It is my guess that, beyond the identification of tissue damage that could account for the a patient’s pain, healthcare practitioners believe patients when:

i) They have known the patient for a long time and regard him or her as a reliable narrator.
ii) They see the patient behave like most patients in a similar situation –that is, in a predictable way ([2]: 8, 201).^32^ Hence the challenge that the “exceptional patient” poses ([1]: 492).
iii) They have had had a similar experience and empathize ([1]: 491).
iv) They have experienced a lack of credibility for an experience that they deemed tough.

This is my suspicion, but only further research could confirm that. In other words, the present inquiry that now reaches its conclusion has focused on the phenomenon of patients with chronic pain *not* being believed. The other side of the phenomenon is those patients with chronic pain who *are* being believed. And the study of the reasons why they are believed, despite their accounts of pain not meeting the conditions of possibility according to the current epistemological model, might help forge –or at least make explicit- an alternative epistemological model that promotes epistemic justice.

## Data Availability

All data generated or analysed during this study are included in this published article.

## Footnotes

1 That form of pain that lasts three months or more ([1]: 490). See Moscoso ([2]: 195 and ff) for a short critical history on the distinction between actue and chronic pain.

2 Jurecic describes the task of medical humanists as follows: “They observe how autobiographical illness narratives reclaim patients’ voices from the biomedical narratives imposed upon them by modern medicine. They study, as well, how literary, popular, and medical narratives report and construct the experience of illness, from the personal level to the national. And they encourage medical practitioners to respond to the stories of suffering people with attention, respect, and understanding” ([6]: 3).

3 Few years before, Moscoso had put it in slightly different words, although Buchman does not quote them: the tension “between subjective experience and the objective knowledge of the illness turns pain into a problem that is at the same time cognitive and moral” ([2]: 166).

4 A lack of diagnosis might also be a *cause* of epistemic injustice, as will be explained in Section 2. Causes of the downgrading of pain sufferers’ credibility.

5 A term coined by sociologist Parsons [32].

6 Often, healthcare workers who “are typically subordinated in the medical hierarchy”, such as “radiation, occupational and physical therapists”, are decisive in helping “patients to restore and reconfigure ruptured identities after serious illness” because of their ability to listen and interact with patients ([34]: 1056).

7 Ultimately, because we tend to assume people’s *reaction* to pain does not always correspond to what they should be *feeling*, either by excess or by default.

8 In turn, those groups that are less listened to, tend to be more interrupted by healthcare practitioners and given less information ([35]: 12). On the other hand, further research is needed to know here if healthcare practitioners who share these same group identities are more understanding and caring than those who do not share the identity.

9 A medicine practiced with the “narrative skills of recognizing, absorbing, interpreting, and being moved by the stories of illness” ([24]: 4) which “will lead to more humane, more ethical, and perhaps more effective care” (vii).

10 That was Arendt’s case, who wrote: “Indeed, the most intense feeling we know of, to the point of blotting out other experiences, namely, the experience of great pain, is at the same time the most private and least communicable of all” ([36]: 51).

11 George, turning to Heidegger and Lévinas, goes as far as to claim that “what Scarry describes as destroyed in pain is not merely the world of the self but the self itself” ([39]: 52), although, in our view, he does not clarify his notion of self.

12 Such praise of metaphors, though, is seldom accompanied by a discussion of what metaphors are. Moreover, they rarely mention the controversy over whether metaphoric language can ultimately be distingished from non-metaphorical language. Against this background, Lunati holds that “the conceptualization of language as continuously metaphorical” is not a minor belief. For her, “first associated with the romantics of the late eighteenth and early nineteenth centuries”, it “has had a much greater acceptance with contemporary philosophers of language such as Nietzsche, Wittgenstein, Foucault, and Derrida, to name but a few” ([41]: 224).

13 Buchman, Ho and Goldberg take the notion of “mechanical objectivity” from Daston and Galison [48]: “This model has two central features: first, knowledge produced via natural investigation has its truth status determined by the extent to which the investigator’s subjective influence is removed from the knowledgemaking process; and second, the primary goal of the investigator is to represent the scientific object just as it appears in its most natural state—no matter how imperfect that object may be in such a state. How does this apply to pain? We can see ideals of mechanical objectivity in the epistemic anxiety that stakeholders voice as to pain. Especially in chronic pain discourse, the natural object does not always reveal the truth of the assertion: the patient’s symptoms do not correlate with any visible scientific object. Thus, pain defies the process of clinical correlation that is central to the anatomo-clinical method” ([10]: 34).

14 Other critics of this type of questionnaire criticize the fact that “[p]atients select from a limited range of words, some of which may not be familiar” ([6]: 49) and that “patients are asked for *words*, not narratives” ([6]: 50).

15 Dumit [50] studied how brain imaging, as long as it reveals that a biological, natural reality is behind an experience, contributes to objectify afflictions and to free patients from feelings of blame and guilt, but for the wrong reasons.

16 In this sense, they align with the growing interest, since at least the 1980s, in illness narratives professed by narratology and anthropology of health.

17 The distinction between illness and disease was popularized in the 1980s, in particular by anthropologists Young [52], Engelhardt [33] and Kleinman.

18 His whole book deploys its argument within this contradiction. As he puts it, “[t]he inexpressibility of pain, then, is our starting point. From there, this book has two main goals: to explore the reasons for this inexpressibility and to discover ways of overcoming them” ([8]: 14).

19 Who precisely studies “what renders pain communicable for some writers, but not for others. Are these differences attributable to the extremity of the pain, the speaker’s or writer’s skill with language, the chosen genre, the audience?” ([6]: 43).

20 A nuance is required in the case of Jackson, author of *Camp Pain* (2000). As Jurecic synthetizes it: “For her, metaphors do not express pain as much as they point to pain’s inexpressibility. Yet, the most vibrant accounts of pain she records involve metaphors” ([6]: 52).

21 For her, metaphors not only describe the experience of pain, but also “have a profound impact on the way we *feel* pain” ([3]: 53).

22 Lunati [41] explores a number of literary works in Catalan concerning illness and, in particular, which metaphors are used and how, with the belief that “positive metaphors can be used by the sick to express the sense of alienation that an illness often brings with it. Finding tropes for one’s illness (whether a real or a fictional one) can be a helpful strategy to come to terms with the representation of that close but noncompliant other” ([41]: 225).

23 Jurecic makes this point à propos Jackson’s work *Camp Pain* (2000): “She acknowledges that patients turn to metaphor to explain their pain, while she argues that it cannot be expressed in everyday language, as if metaphor were not, in fact, part of ordinary language” ([6]: 52). The same critique could be made to Biro’s work, which, in part, is an arduous attempt to hold that metaphor is more appropriate than ordinary language to convey the experience of pain. His whole work rests upon this premise: “there is only one way to open us: metaphor (…), a powerful and necessary resource of the imagination that literally extends the boundaries of our shared world” ([8]: 16). But his work seems problematic insofar as it does not problematize the blurry threshold between metaphor and ordinary language, which philosophy of language has extensively studied. Lunati’s escapes this problem because, in spite of underlining the capacity metaphors have to express illness, she adheres to Derrida’s view according to which, ultimately, all language is metaphorical.

24 And so does Moscoso [2] in the fifth chapter of his cultural history of pain.

25 It is a tendency, but not a generalized practice. Many patients with pain that does not have an identifiable cause are, indeed, believed –i.e., patients with headache, neuropathes or back pain which seem to have no explanation are often believed. But the lack of an objectively-viewed cause increases the risk of healthcare professionals not regarding that form os pain as valid.

26 Moreover, this epistemological frame cannot fully account for those pathologies in which the very subjectivity of the individual is at stake, as is often the case for mental illnesses.

27 For her, the very act of naming an experience “pain” influences the experience: “Once taught what constitutes a pain-event, subtle messages communicated through language, facial expressions, and gestures help inform people-in-pain how they ought to respond when registering it (…) These communicative acts are normative” ([3]: 17).

28 On the one hand, because, for being in pain, it is enough to claim that one is in pain. It is as if, in the case of pain, what grants objectivity is subjectivity –the distinction collapses. But, on the other hand, as our analysis of Bourke’s work concludes, pain seems to be something objective that we all experience subjectively –that is, the phenomenon of pain cannot be accounted for without this distinction.

29 Secondary gain is described as “the advantage that occurs secondary to stated or real illness. Transition into the sick role may have some incidental secondary gains for patients. Types of secondary gain include using illness for personal advantage, exaggerating symptoms, consciously using symptoms for gain, and unconsciously presenting symptoms with no physiological basis. These symptoms may contribute to the social breakdown syndrome and the patient's choice to remain in the sick role” ([56]: 10).

30 And he goes on as follows: “Until the (…) narrative can be honored, the world in all its possibilities is being denied. To deny (…) story is to deny the person telling this story, and people who are being denied cannot be cared for” ([23]: 109). That it, listening to and believing a patient not only has important ethical and existential consequences, but also tends to derive in a relationship of trust between patients and professionals that leads to a better pain assessment and subsequent management –in short, to a most effective care [1, 18, 20].

31 The existing literature suggests that is not always a matter of verbal recognition and decisions concerning further treatment, but also includes enacting “simple actions of comfort. As a patient walks into the clinic, a steadying hand, a chair placed in a more convenient position or permission to stand if more comfortable, will give the patient confirmation and ratify non-verbally that his/her pain is being accepted at face value” ([1]: 493).

32 This might be one of the reasons why, as the literature on the topic highlights, patients try to “produce” the common symptoms associated with the pathology they think they have. When making this point, Clarke and Iphofen recall a study [19] which concluded “that the women all tried to conform to what they perceived they thought the doctor would want to see, and did not want to be considered as whiners or complainers” ([1]: 491).
